# Total Flavonoids from *Carthamus tinctorius* L. Reduce Liver Fibrosis by Influencing Autophagy via Hedgehog Signaling

**DOI:** 10.3390/ijms27135957

**Published:** 2026-07-02

**Authors:** Rui Yang, Mingqi Li, Chenlu Zhang, Yinghe Wang, Shuangjing Zhang, Huijun Liang, Liyan Sun, Rong Jin, Xiaomei Bao, Yuehong Ma

**Affiliations:** 1 School of Basic Medicine, Inner Mongolia Medical University, Hohhot 010010, China; 2 Key Laboratory of Molecular Pathology of Inner Mongolia Autonomous Region, School of Basic Medicine, Inner Mongolia Medical University, Hohhot 010110, China; 3 School of Pharmacy, Inner Mongolia Medical University, Hohhot 010110, China

**Keywords:** TFCTL, liver fibrosis, HSC-T6, Hedgehog signaling, autophagy

## Abstract

Liver fibrosis is a critical determinant of the progression of chronic liver disease (CLD). Total flavonoids from *Carthamus tinctorius* L. (TFCTLs) exhibit diverse pharmacological activities while their effect on liver fibrosis remains incompletely understood. This study aimed to elucidate the effects and mechanisms of TFCTLs on liver fibrosis. To this end, we first established a carbon tetrachloride (CCl_4_)-induced liver fibrosis model in mice. Histological analysis demonstrated that TFCTL treatment significantly alleviated CCl_4_-induced liver collagen deposition (*p* < 0.001). Meanwhile, TFCTLs could also downregulate the expression levels of fibrosis markers α-SMA and collagen I in a dose-dependent manner (*p* < 0.05). In vitro, a cellular model of fibrosis was generated by treating HSC-T6 cells with TGF-β1. EdU incorporation assays revealed that TFCTLs significantly suppressed HSC proliferation (*p* < 0.05). Furthermore, immunofluorescence staining for α-SMA demonstrated a marked reduction in HSC activation upon TFCTL treatment. The inhibitory effect of TFCTLs on cell migration was confirmed by wound healing and transwell assays, which revealed a substantial decrease in the number of migrated cells (*p* < 0.001). Additionally, flow cytometric analysis indicated that TFCTL treatment promoted HSC apoptosis (*p* < 0.05). Further mechanistic investigations revealed that TFCTLs exert their antifibrotic effects by inhibiting Hedgehog pathway and activating autophagy process. The antifibrotic effect of TFCTLs was partially reversed by the autophagy inhibitor 3-MA. Furthermore, the Hedgehog agonist PUR not only counteracted the anti-fibrotic actions of TFCTLs but also suppressed TFCTL-induced autophagy activation. In conclusion, our study demonstrated that TFCTLs attenuate liver fibrosis by inhibiting Hedgehog signaling and subsequently promoting autophagy, highlighting their potential as a therapeutic agent for liver fibrosis.

## 1. Introduction

Liver fibrosis, a core pathological hallmark of chronic liver disease, results from persistent liver injury caused by various etiologies, including viral hepatitis, cholestasis, alcohol consumption, metabolic disturbances, and autoimmune conditions [1]. Although the early stage of liver fibrosis is reversible, advanced fibrosis often progresses to irreversible conditions such as cirrhosis and liver failure [2]. A key feature of liver fibrosis is the excessive accumulation of extracellular matrix (ECM) [3,4], primarily produced by activated hepatic stellate cells (HSCs) [5]. In response to sustained liver injury, HSCs activate and transdifferentiate into myofibroblasts (MFBs), characterized by enhanced proliferation, contractility, inflammation, and chemotaxis. This process is accompanied by a significant increase in α-smooth muscle actin (α-SMA) expression and leads to the aberrant deposition of ECM components such as type I collagen (collagen I), thereby establishing a vicious cycle that perpetuates fibrogenesis [6]. Therefore, suppression of HSC activation is considered a major therapeutic target for treatment of liver fibrosis.

Recent studies have implicated autophagy in HSC activation, although its precise role remains controversial. In some contexts, autophagy promotes HSC activation and exacerbates fibrosis [7]. However, several studies have shown that activation of HSC autophagy inhibits extracellular vesicle release to attenuate liver fibrosis [8,9,10,11,12]. Therefore, targeting HSC autophagy may represent a promising therapeutic strategy for liver fibrosis.

In recent years, traditional Chinese medicines (TCMs) have demonstrated distinct advantages in the prevention and treatment of liver fibrosis. Total flavonoids from *Carthamus tinctorius* L. (TFCTLs) represent the flavonoid-rich fraction derived from safflower, a well-known medicinal herb. Our previous study revealed that TFCTLs inhibit the proliferation and activation of hepatic stellate cells (HSCs) through modulation of the Hippo/YAP signaling pathway [13]. Given that TCMs typically exert their therapeutic effects through multi-target interactions, in the present study, we sought to determine whether TFCTLs exert their anti-fibrotic effects through modulation of HSC autophagy, and to further elucidate the underlying molecular mechanisms.

The Hedgehog (Hh) signaling pathway is a classical cascade comprising Hh ligands, the receptor Patched (Ptch), the signal transducer Smoothened (SMO), and downstream transcription factors of the Gli family (Gli1/2/3) [14]. Recent studies have highlighted its regulatory role in liver regeneration and injury repair [15]. During hepatic fibrogenesis, injured hepatocytes release Hh ligands that bind to Ptch1 receptor on HSCs, thereby activating SMO, and promoting Gli1 nuclear translocation, which in turn drives the transdifferentiation of HSCs into myofibroblasts (MFBs) [16,17]. Activation of Hh signaling enhances HSC proliferation and ECM synthesis [18]. Our previous analysis revealed that the Hh pathway is closely associated with TFCTLs suggesting that the Hh pathway may serve as a potential target through which TFCTLs regulate HSC autophagy.

## 2. Results

### 2.1. TFCTLs Ameliorate Hepatic Fibrosis in Mice

**Figure 1:**
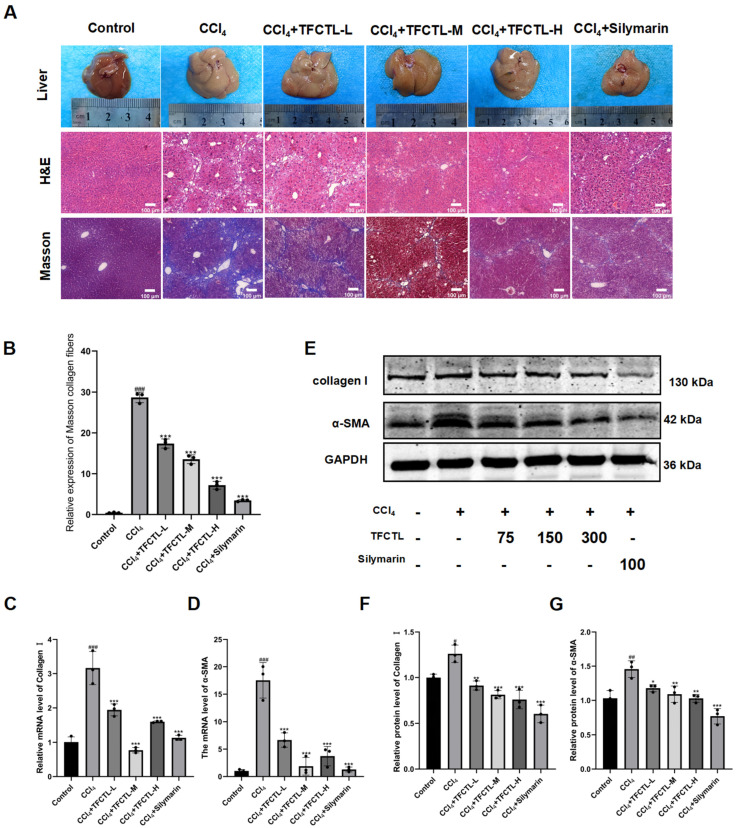
**Effects of TFCTLs on liver fibrosis:** (**A**) Gross appearance, H&E staining, and Masson staining results of liver tissues (200×; scale bar: 100 μm). (**B**) Statistical graph of the collagen fiber content in liver tissues from each group. (**C**,**D**) mRNA expression levels of α-SMA and collagen I in liver tissues detected by RT-qPCR. (**E**–**G**) Protein expression levels of collagen I and α−SMA detected by Western blot. GAPDH is used as a loading control (*n* = 3, # *p* < 0.05, ## *p* < 0.01, ### *p* < 0.001 vs. control; * *p* < 0.05, ** *p* < 0.01, *** *p* < 0.001 vs. CCl_4_ group).

First, we investigated the therapeutic effect of total flavonoids from *Carthamus tinctorius* L. (TFCTLs) on hepatic fibrosis in mice. We administered low, medium, and high doses of TFCTLs (75, 150, and 300 mg/kg, respectively); silymarin was used as a positive drug control. As shown in Figure 1A, livers of control group had smooth, bright red surfaces and were soft in texture. In contrast, mice in the model group exhibited significant pathological changes, including increased surface roughness, irregular borders, dull color, slight swelling, and a granular texture but application of low, medium, and high doses of TFCTLs and silymarin presented more glossy liver surfaces and improved border regularity (Figure 1A). H&E staining revealed that the liver lobule structure was disrupted in the model group, with evident fibrous septum formation, hepatocyte swelling accompanied by lipid vacuolation, and substantial inflammatory cell aggregation in the portal areas. Compared with the model group, the TFCTL treatment groups and the Silymarin group exhibited significantly alleviated liver structural disorganization, a reduced density of fibrous septa, and decreased areas of cell necrosis. The results of Masson staining further demonstrated that, compared with the model group, all the TFCTL treatment groups and the Silymarin group effectively inhibited excessive collagen deposition, with the number of collagen fibers in the portal areas significantly reduced (Figure 1B). Furthermore, the RT–qPCR results revealed that the mRNA expression levels of the hepatic fibrosis markers α-SMA and collagen I were significantly increased in the CCl_4_-induced model group. In contrast, these levels were significantly decreased in the low-, medium-, and high-dose TFCTL groups and the Silymarin group than in the CCl_4_-induced model group (Figure 1C,D). Western blot results showed that the protein expression levels of α-SMA and collagen I were also significantly lower in the low medium, and high-dose TFCTL groups and the Silymarin group than in the CCl_4_ group (Figure 1E–G). These results indicate that TFCTLs have a significant anti-hepatic fibrosis effect in vivo.

### 2.2. TFCTLs Inhibit HSC Activation, Proliferation, and Migration to Ameliorate Liver Fibrosis

**Figure 2:**
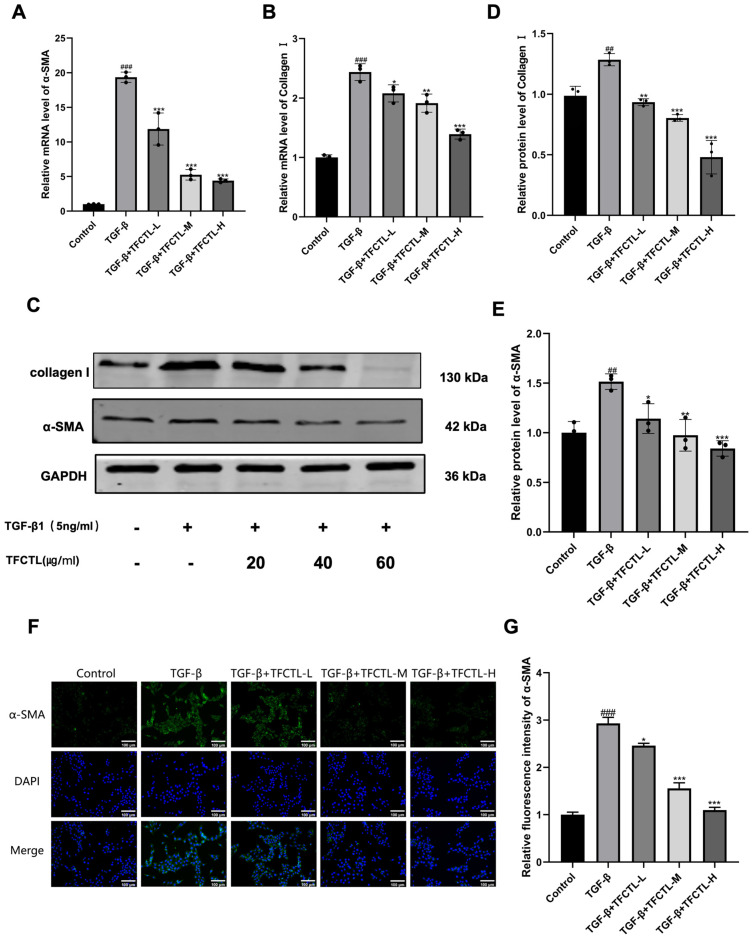
**TFCTLs inhibit the expression of fibrosis-related markers in hepatic stellate cells in vitro:** (**A**,**B**) RT-qPCR assays are performed to measure the mRNA expression levels of α-SMA and collagen I. (**C**–**E**) The protein expression levels of α-SMA and collagen I are measured, and GAPDH is used as a loading control. (**F**,**G**) The expression level of α-SMA is detected by immunofluorescence (20×; scale bar: 100 µm) (*n* = 3, ## *p* < 0.01, ### *p* < 0.001 vs. the control; * *p* < 0.05, ** *p* < 0.01, *** *p* < 0.001 vs. TGF-β group).

**Figure 3:**
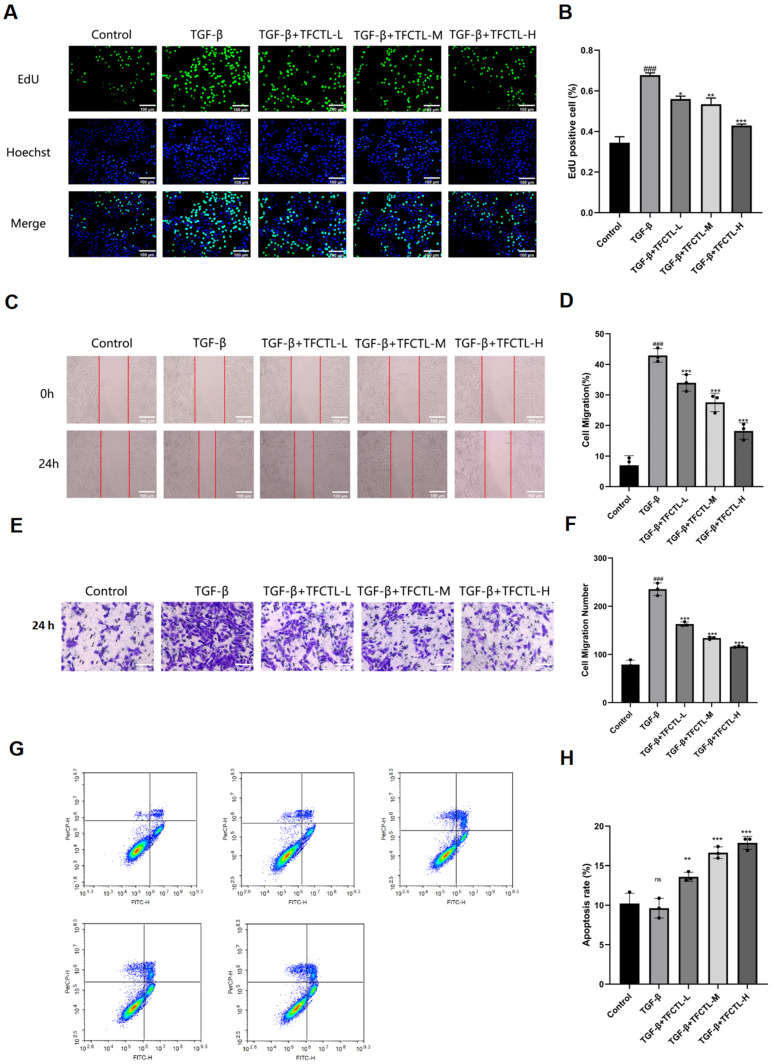
**TFCTLs suppress proliferation and migration, and induce apoptosis in hepatic stellate cells:** (**A**,**B**) Cell proliferation rates are detected by an EdU assay (20×, scale bar: 100 µm). (**C**,**D**) Cell migration is detected by a wound healing assay (20×; scale bar: 100 µm). (**E**,**F**) Transwell assays are performed to confirm the cell migration rates (20×; scale bar: 100 µm). (**G**,**H**) The apoptosis rate is detected by flow cytometry assays (TFCTL-L: TGF-β1 + 20 μg/mL TFCTL-L; TFCTL-M: TGF-β1 + 40 μg/mL TFCTL-M; TFCTL-H: TGF-β1 + 60 μg/mL TFCTL-H) (*n* = 3, ### *p* < 0.001 vs. the control; * *p* < 0.05, ** *p* < 0.01, *** *p* < 0.001 vs. TGF-β group, ns: not significant).

Numerous studies have shown that hepatic stellate cell (HSC) activation and proliferation are closely associated with the development of liver fibrosis [19,20]. Therefore, to further elucidate the effect of TFCTLs on liver fibrosis, we conducted experiments using rat hepatic stellate cells (HSCs-T6) in vitro. RT–qPCR and Western blot results demonstrated that TGF-β1 treatment significantly upregulated the mRNA and protein expression levels of the fibrosis-related markers α-SMA and collagen I. In contrast, treatment with TFCTLs significantly reduced the TGF-β1-induced expression of α-SMA and collagen I (Figure 2A–E). Immunofluorescence staining revealed that α-SMA expression was significantly increased in the TGF-β1 group, confirming that TGF-β1 enhances HSC-T6 activation. In addition, treatment with different concentrations of TFCTLs (20, 40, and 60 μg/mL) significantly reduced α-SMA expression, indicating that TFCTLs effectively inhibited HSC-T6 activation (Figure 2F,G). Furthermore, to confirm the effect of TFCTLs on cell proliferation, migration and apoptosis, the EdU staining was first performed. As shown in Figure 3A, compared with the control group, the TGF-β1-induced group exhibited a significant increase in cell proliferation activity while after intervention with different concentrations of TFCTLs, the cell proliferation rates in the low-, medium-, and high-dose groups were significantly decreased compared with those in the TGF-β1 group (Figure 3B). Additionally, the results of wound healing and Transwell assays revealed that TFCTL treatment inhibited TGF-β1-induced cell migration (Figure 3C–F). Moreover, the flow cytometry results indicated that compared with the TGF-β1 group, all the TFCTL treatment groups had significantly increased apoptosis in the HSC-T6 cells (Figure 3G,H). These findings collectively demonstrate that TFCTLs can ameliorate liver fibrosis by inhibiting the proliferation, activation, and migration and promoting the apoptosis of hepatic stellate cells.

### 2.3. TFCTLs Ameliorate Hepatic Fibrosis by Targeting the Hedgehog Signaling Pathway

**Figure 4:**
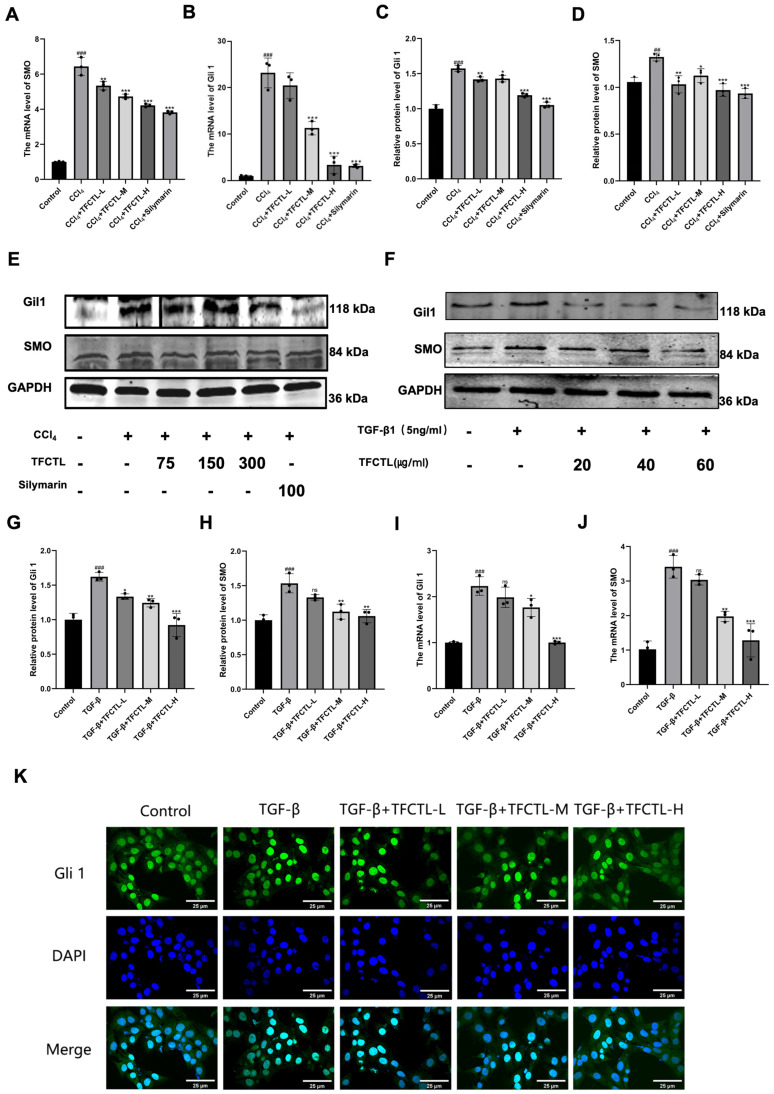
**Effects of TFCTLs on the Hh signaling pathway:** (**A**,**B**) RT–qPCR assays are performed to detect the mRNA expression levels of Gil1 and SMO in liver tissues. (**C**–**E**) Protein expression levels of Gil1 and SMO; GAPDH is used as a loading control. (**F**,**G**) RT–qPCR assays are performed to detect the mRNA expression levels of Gil1 and SMO in HSCs. (**H**–**J**) Protein expression levels of Gil1 and SMO; GAPDH is used as a loading control. (**K**) Immunofluorescence assays are performed to verify the nuclear expression of Gli1 (scale bar: 25 µm) (*n* = 3, ## *p* < 0.01, ### *p* < 0.001 vs. the control; * *p* < 0.05, ** *p* < 0.01, *** *p* < 0.001 vs. TGF-β group, ns: not significant).

In our previous study, we employed network pharmacology to explore the potential mechanisms through which TFCTL exert their antifibrotic effects. KEGG pathway enrichment analysis revealed a strong association between the Hedgehog (Hh) signaling pathway and TFCTLs. Recently, numerous studies have shown that Hh signaling is involved in fibrotic diseases, but its role in liver fibrosis is not very unclear. Therefore, we hypothesized that TFCTL might exert its antifibrotic effects by modulating the Hh signaling pathway. SMO and Gli1 are a downstream receptor and transcription factor of Hh signaling and are upregulated during Hh activation. Thus, we first detected the mRNA and protein expression levels of SMO and Gli1 by Western blot and RT–qPCR. Compared with the control group, the CC model group exhibited significantly increased expression levels of both SMO and Gli1. However, the administration of low, medium, and high doses of TFCTLs, as well as the positive control drug Silymarin, effectively reversed these changes, leading to pronounced decreases in SMO and Gli1 expression at both the mRNA and protein levels (Figure 4A–E). Consistent with the in vivo results, treatment with TGF-β1 could also have significantly upregulated the mRNA and protein expression levels of SMO and Gli1 (Figure 4F–J). This induction was markedly suppressed by treatment with various concentrations of TFCTL, with the most potent inhibitory effect observed in the high-dose group (*p* < 0.05). Given that Hh pathway activation is characterized by the increased nuclear translocation of Gli1, we then further used immunofluorescence to detect the nuclear expression of Gli1. As shown in Figure 4K, the TGF-β1-treated group displayed significant accumulation of Gli1 within the nucleus, as evidenced by increased nuclear fluorescence intensity. In contrast, compared with the TGF-β1 group, all the TFCTL treatment groups presented a substantial reduction in nuclear fluorescence intensity, further suggesting the inhibition effect of TFCTLs on the Hh pathway. In conclusion, our findings demonstrate that TFCTLs alleviate hepatic fibrosis by inhibiting the activation of the Hedgehog signaling pathway in HSCs, primarily through the downregulation of SMO and Gli1 expression and the suppression of Gli1 nuclear translocation.

### 2.4. TFCTLs Alleviate Hepatic Fibrosis by Activating Autophagy

**Figure 5:**
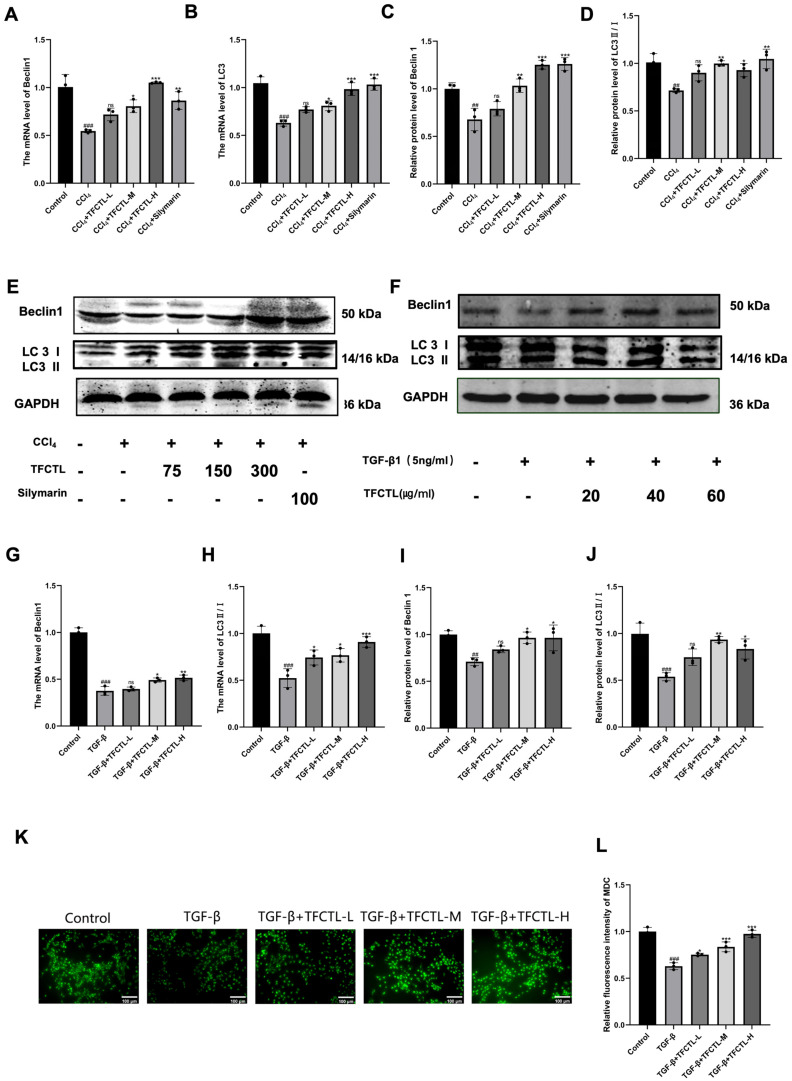
**Effects of TFCTLs on autophagy:** (**A**,**B**) RT–qPCR assays are performed to detect the mRNA expression levels of Beclin1 and LC3 in liver tissues. (**C**–**E**) Protein expression levels of Beclin1 and LC3; GAPDH is used as a loading control. (**F**,**G**) RT–qPCR assays are performed to detect the mRNA expression of Beclin1 and LC3 in HSCs. (**H**–**J**) Protein expression levels of Beclin1 and LC3; GAPDH is used as a loading control. (**K**,**L**) MDC staining is performed to measure the level of autophagy (scale bar: 100 µm) (*n* = 3, ## *p* < 0.01, ### *p* < 0.001 vs. the control; * *p* < 0.05, ** *p* < 0.01, *** *p* < 0.001 vs. TGF-β group, ns: not significant).

Activation of Hh signaling is always correlated with a reduction in autophagy in fibroblasts, which may further promote fibrosis [21]. Thus, we sought to further investigate whether TFCTLs could also modulate autophagy during liver fibrosis. RT–qPCR and Western blot analysis revealed that compared with those in the control group, the expression levels of the autophagy-related markers LC3 and Beclin1 were significantly downregulated at both the mRNA and protein levels in the CCl_4_-induced model group, indicating that autophagy was inhibited during liver fibrosis (Figure 5A–E). In contrast, treatment with different concentrations of TFCTLs markedly upregulated their expression, thereby promoting autophagy activation, with the medium- and high-dose groups showing the most pronounced effects. Consistent with these findings, in vitro experiments also demonstrated that administration of TFCTLs resulted in notable increases in the mRNA and protein expression levels of LC3 and Beclin1. Moreover, medium and high concentrations of TFCTLs significantly increased the expression of these genes at both the transcriptional and translational levels (Figure 5F–J).

To further visualize autophagic activity, we employed monodansylcadaverine (MDC), a fluorescent probe widely used to label autophagosomes due to its ion-trapping and membrane phospholipid-binding properties. MDC staining revealed that TGF-β1 treatment weakened the relative fluorescence intensity in HSCs. In contrast, TFCTL treatment resulted in stronger green fluorescent puncta in HSC-T6 cells, accompanied by a statistically significant increase in relative fluorescence intensity, indicating an increased number of autophagosomes (Figure 5K–L). In summary, these results demonstrate that TFCTLs alleviate liver fibrosis by activating autophagy in hepatic stellate cells.

### 2.5. TFCTLs Suppress Hepatic Stellate Cell Activation by Inhibiting the Hedgehog Signaling Pathway and Activating Autophagy

**Figure 6:**
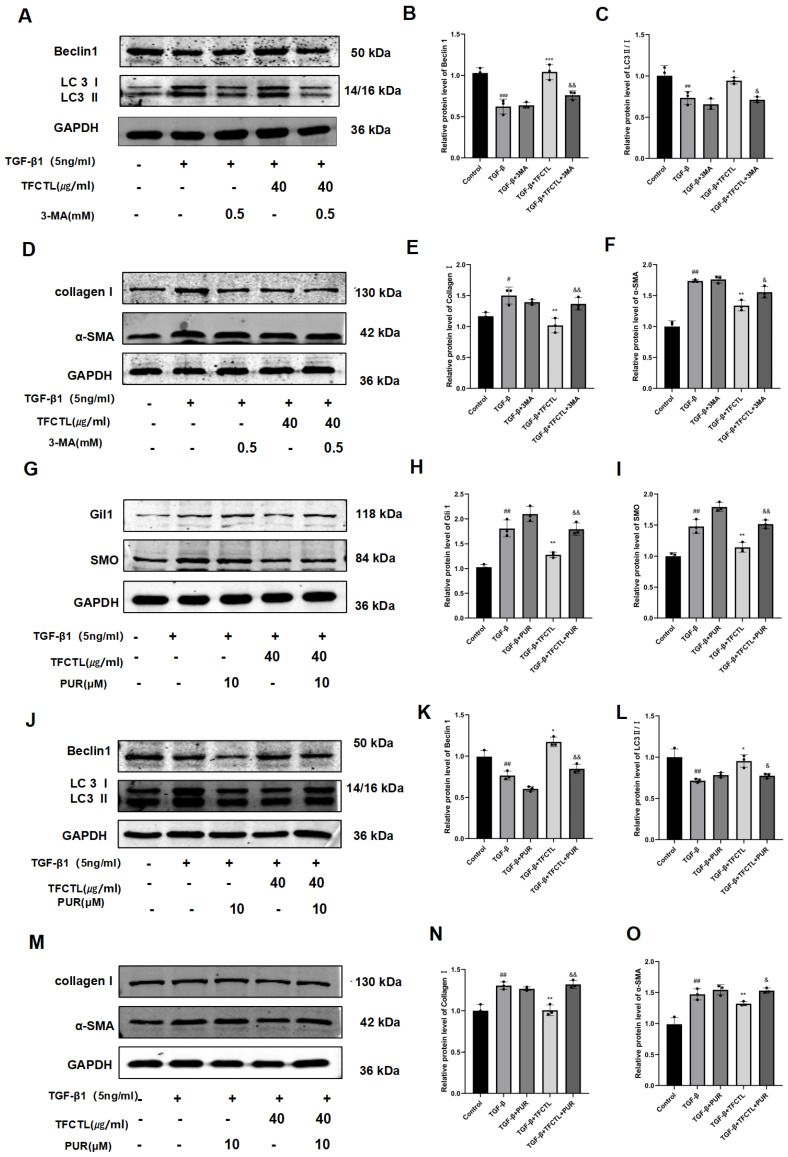
**Activation of Hh signaling reversed the antifibrotic effect of TFCTL:** (**A**–**C**) Protein expression levels of Beclin1 and LC3; GAPDH is used as a loading control; 3-MA: autophagy inhibitor, 0.5 mM. (**D**–**F**) Protein expression levels of α-SMA and collagen I; GAPDH is used as a loading control. (**G**–**I**) Protein expression levels of Gil1 and SMO; GAPDH is used as a loading control; PUR: Hh activator, 10 µM. (**J**–**L**) Protein expression levels of Beclin1 and LC3. (**M**–**O**) Protein expression levels of α-SMA and collagen I (*n* = 3, # *p* < 0.05, ## *p* < 0.01, ### *p* < 0.001 vs. the control; * *p* < 0.05, ** *p* < 0.01, *** *p* < 0.001 vs. TGF-β group; and & *p* < 0.5, && *p* < 0.01 vs. TGF-β + TFCTL group).

To further elucidate the mechanism by which TFCTLs ameliorate hepatic fibrosis through targeting the Hh signaling pathway and autophagy, we at first treated HSC-T6 cells in vitro with the autophagy inhibitor 3-MA (0.5 mM). Western blot analysis revealed that compared with TFCTL alone, the combination of TFCTL and 3-MA (TGF-β1 + TFCTL + 3-MA) significantly increased the expression levels of the autophagy-related proteins LC3-II/I and Beclin1, indicating that 3-MA reversed the TFCTL-induced activation of autophagy in activated HSC-T6 cells (Figure 6A–C). Moreover, compared with the TFCTL-treated group, the TGF-β1 + TFCTL + 3-MA group exhibited markedly upregulated expression levels of the fibrosis-related markers α-SMA and collagen I, suggesting that 3-MA counteracted the inhibitory effect of TFCTLs on HSC-T6 activation (Figure 6D–F). These results suggest that TFCTLs suppress HSC activation by inducing autophagy, thereby contributing to their antifibrotic effects.

**Figure 7:**
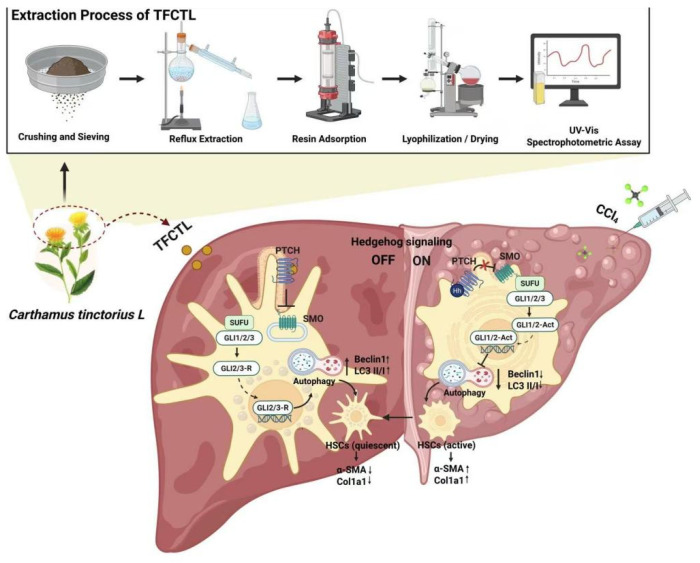
**TFCTLs inhibit liver fibrosis by targeting Hh signaling and activating autophagy.** Our study finds that TFCTLs exerted excellent anti-liver fibrosis effects in a dose-dependent manner by inhibiting the Hedgehog (Hh) signaling pathway, and subsequently activating autophagy, which in turn attenuates hepatic stellate cell activation and proliferation while promoting apoptosis (↓: decreased; ↑: increased).

We subsequently treated HSCs with the Hedgehog signaling agonist PUR (10 μM). Compared with those in the TGF-β1 + TFCTL group, the expression levels of the Hh signaling-related molecules SMO and Gli1 significantly increased in the TGF-β1 + TFCTL + PUR group (Figure 6G–I). Concurrently, the expression levels of the autophagy-related proteins LC3-II/I and Beclin1 were also notably elevated in the PUR cotreatment group, indicating that activation of the Hh pathway reversed the inductive effect of TFCTLs on autophagy (Figure 6J–L). Furthermore, the Western blot results also demonstrated that compared with the TGF-β1 + TFCTL treatment, the addition of PUR led to significant increases in the expression levels of the HSC activation markers α-SMA and collagen I (Figure 6M–O). In summary, these findings support the conclusion that TFCTLs alleviate hepatic fibrosis by inhibiting the Hedgehog signaling pathway and subsequently promoting autophagy in hepatic stellate cells (Figure 7).

## 3. Discussion

In this study, we found that total flavonoids from *Carthamus tinctorius* L (TFCTL) could inhibit the activation and proliferation of hepatic stellate cells and subsequently protect against liver fibrosis. In recent years, the ability of natural nutrients and products to prevent and treat liver fibrosis has attracted widespread attention. Herpetrione, an active lignan isolated from *Herpetospermum pedunculosum*, was determined to ameliorate liver injury and fibrosis by targeting FXR [22]. Ergothioneine, derived from certain edible fungi, can suppress hepatic stellate cell activation and improve CCl4-induced liver fibrosis and liver injury [23,24]. In addition, silymarin, a flavonolignan-rich extract of *Silybum marianum*, is widely recognized for its hepatoprotective potential [25,26,27], suggesting that total flavonoids derived from natural plants have the potential to protect the liver.

*Carthamus tinctorius* L (safflower) is a widely used herb in traditional Chinese medicine. According to traditional Chinese medicine (TCM) theory, safflower functions to invigorate blood circulation, dispel stasis, and regulate menstruation, thereby alleviating associated pain [28]. Recent studies have shown that, in addition to their promising anti-inflammatory effects [29], safflower extracts and their nanoparticles possess high potential activity as antimicrobial, antioxidant, and anticancer agents [30,31]. Additionally, safflower has shown promise in combating anxiety and depression with marked effectiveness [32].

Recent research has revealed that safflower possesses a variety of chemical constituents that exhibit a wide range of pharmacological activities, underscoring its therapeutic potential. Currently, more than 104 compounds from safflower have been isolated and identified, including polysaccharides, flavonoids, and alkaloids [28,33]. Phytochemical studies have revealed that flavonoids are the primary bioactive constituents in safflower. These compounds, including quinochalcones (e.g., hydroxysafflor yellow A and carthamin) and flavonols (e.g., kaempferol and quercetin derivatives), demonstrate a spectrum of biological activities, such as dilating coronary arteries, improving myocardial ischemia, and modulating immune responses [34,35,36,37]. Recent studies have indicated that safflower flavonoid extracts exhibit significant hepatoprotective effects and play important roles in treating conditions such as nonalcoholic fatty liver disease and liver injury. Quercetin and kaempferol from safflower play crucial roles in combating NAFLD by mitigating hepatocyte steatosis [38]. Hydroxysafflor yellow A alleviates oxidative stress and inflammatory damage in the livers of mice with nonalcoholic fatty liver disease (NAFLD) by modulating the gut microbiota [39] and protecting against alcoholic liver diseases through multiple targets [40]. Furthermore, HSYA has been reported to inhibit CCl4-induced liver fibrosis [41]. Although more than 60 flavonoids have been identified in safflower [42], most existing studies focus on individual compounds, leaving the overall hepatoprotective effect and underlying mechanisms of total flavonoids from *Carthamus tinctorius* L (TFCTL) largely unknown. Our preliminary research, which utilized network pharmacology and transcriptome sequencing, revealed the significant liver-protecting potential of TFCTL. Therefore, this study is designed to investigate its specific therapeutic effects against liver fibrosis.

To further investigate the regulatory role and mechanism of TFCTLs in liver fibrosis, we first established an in vivo liver fibrosis model. CCl4, a hepatotoxic substance, effectively induces liver fibrosis in rodents [43,44]. We found that the CCl4-induced model group exhibited significant collagen deposition and upregulated the expression levels of fibrosis-related markers in liver tissue. In contrast, treatment with low, medium, and high doses of TFCTLs significantly alleviated the degree of liver fibrosis in a dose-dependent manner. Hepatic stellate cell activation is the key mechanism leading to fibrosis [45]. Promoting the apoptosis of HSCs and reducing their activation are essential therapeutic strategies for the management of liver fibrosis. To this end, we investigated the effects of different concentrations of TFCTLs on the activation and apoptosis of TGF-β1-induced hepatic stellate cells (HSCs) in vitro. Our results showed that TFCTLs significantly inhibited TGF-β1-induced HSC activation, proliferation, and migration while promoting apoptosis. These findings demonstrate that TFCTLs can significantly attenuate liver fibrosis.

Our preliminary bioinformatics analysis revealed that the Hedgehog (Hh) signaling pathway may be a key target through which TFCTLs intervene in liver fibrosis. Hedgehog (Hh) signaling is evolutionarily conserved and plays an instructional role in embryonic development and tissue homeostasis [46,47]. Hedgehog (Hh) proteins constitute a family of a small number of secreted signaling proteins that together regulate multiple aspects of diseases such as malignant cancer [48]. Inhibiting the Hh pathway ameliorates alcohol-associated liver injury in mice [49], and excessive activation of the Hh pathway promotes the development of NASH, cirrhosis, and primary liver cancer [50]. Moreover, numerous studies have shown that dysregulated Hh signaling is involved in fibrotic diseases, and some Hh-targeted inhibitors are currently under exploration in preclinical and clinical trials as a means to prevent fibrosis progression [51]. Thus, we hypothesized that TFCTLs might exert their antifibrotic effect by inhibiting Hh signaling. SMO and Gli1 are downstream targets of Hh signaling [52,53]. We found that the expression levels of SMO and Gli1 were significantly increased during liver fibrosis, indicating the activation of Hh signaling during hepatic fibrosis. However, the application of TFCTLs decreased the expression of SMO and Gli1. Collectively, these findings demonstrate that the antifibrotic effect of TFCTLs is mediated through the inhibition of the Hedgehog (Hh) signaling pathway.

Activation of Hh signaling is always correlated with a reduction in autophagy in fibroblasts, which may further promote fibrosis [21]. Furthermore, a recent study revealed that restoring autophagy alleviates liver fibrosis [17]. Therefore, we first assessed autophagic activity during liver fibrosis. We found that autophagy was significantly suppressed during fibrosis both in vitro and in vivo, whereas the application of TFCTLs reversed this effect. Notably, the protective effect of TFCTLs was abolished upon coadministration of the autophagy inhibitor 3-MA, indicating that TFCTLs mitigate fibrosis by activating autophagy.

To further elucidate whether TFCTLs target the Hh pathway to regulate autophagy, we coadministered the Hh agonist PUR with TFCTLs in HSCs. This combination reversed both the antifibrotic and the proautophagic effects of TFCTLs. Our findings demonstrate that total flavonoids from *Carthamus tinctorius* L. (TFCTL) exert their antifibrotic effects by inhibiting the Hedgehog signaling pathway, which in turn activates autophagy. This cascade ultimately reduces the activation, proliferation, and migration of hepatic stellate cells while promoting their apoptosis.

This study systematically elucidates the effects and mechanisms of total flavonoids from *Carthamus tinctorius* L. (TFCTL) against liver fibrosis. We confirmed that TFCTLs exert their antifibrotic effect by targeting the Hedgehog (Hh) signaling pathway. However, several limitations of the present study should be acknowledged. As TFCTL is a multicomponent extract, the specific flavonoid constituents responsible for its primary efficacy remain to be further identified. In addition, our preliminary investigations revealed that compared with total flavonoid extract, individual flavonoid constituents exhibit weaker antifibrotic activity, suggesting potential synergistic interactions among the components. We thus propose a new scientific hypothesis: certain flavonoids within TFCTL can form supramolecular assemblies with enhanced stability and lip solubility, thereby contributing to its superior therapeutic efficacy—a direction that will be the focus of our future research. In addition, although we have demonstrated a functional link between the Hedgehog (Hh) signaling pathway and autophagy in hepatic stellate cells (HSCs) during fibrogenesis, the precise molecular crosstalk between these two pathways—particularly the hierarchical relationship and the specific signaling nodes involved—remains to be fully elucidated. Finally, although our toxicological evaluation in both male and female mice confirmed a favorable safety profile, the present efficacy study was limited to male animals. Future investigations incorporating both sexes are needed to validate the sex-independent efficacy of TFCTLs and to fully account for sex as a biological variable. Addressing these questions will provide a more solid foundation for its potential clinical application in liver fibrosis.

## 4. Methods and Materials

### 4.1. TFCTL Extraction, Quality Control, and Dose Preparation

The extraction method for TFCTLs was similar to that described in our previous study [13]. In brief, Safflower (*Carthamus tinctorius* L.) l (No. C294220505) was purchased from Anguo Ronghua Herbal Medicine Co., Ltd. (Anguo, China). The dried plant material was pulverized, sieved, and extracted twice with 10 volumes (*v*/*w*) of 80% ethanol under reflux for 2h and 1h, respectively. The two filtrates were combined, concentrated under reduced pressure, and suspended in distilled water. The suspension was then subjected to macroporous resin (AB-8) column chromatography, eluted with 95% ethanol, and the eluate was collected, concentrated, and lyophilized to obtain TFCTL powder. The total flavonoids from *Carthamus tinctorius* L. are stored in Laboratory of School of Pharmacy, Inner Mongolia Medical University (specimen number: 20230030).

TFCTL was determined spectrophotometrically at 510 nm using rutin as the reference standard. A standard curve was constructed (regression equation: Y = 32.45x − 0.0034, R^2^ > 0.999), and the flavonoid content in the extract was calculated to be 60%. Then, the TFCTL powder was formulated at three doses—75, 150, and 300 mg·kg^−1^—based on the dose conversion table of equivalent surface area between humans and animals.

### 4.2. Animals

A total of 72 male C57BL/6 mice (six to eight weeks old) were purchased from the Experimental Animal Center of Inner Mongolia Medical University and reared in a standard animal feeding environment according to the regulations of the Institutional Animal Care and Use Committee of Inner Mongolia Medical University (YKD2019144). The mice were then randomly divided into six groups of 12 mice each: control, model, high, medium and low doses of total flavonoids from the *Carthamus tinctorius* L (TFCTL) groups (300, 150, and 75 mg/kg) and silymarin (100 mg/kg) as a positive control group. Liver fibrosis was induced by the intragastric administration of 2 mL/kg (20% olive oil dilution concentration) carbon tetrachloride (CCl_4_) for 8 weeks twice a week. The drugs were dissolved in 0.5% CMC-Na, and the control and model groups were given 0.5% CMC-Na to eliminate the influence of the solvent. At the end of the experiment, the mice were sacrificed, after which the livers were collected.

### 4.3. Cell Culture

HSC-T6 cells were cultured in high-glucose DMEM supplemented with 10% FBS and 1% penicillin–streptomycin and placed in a constant-temperature incubator with 5% CO_2_ at 37 °C until they were subcultured. HSC-T6 cells were treated with TGF-β1 (5 ng/mL) for 24 h. The cells were subsequently treated with different concentrations of TFCTL (20, 40, or 60 μg/mL).

### 4.4. Immunohistochemistry

The liver tissue was fixed in 4% neutral paraformaldehyde for 48 h. Fixed liver samples were subsequently dehydrated with ethanol (at concentrations of 70%, 80%, and 90%), cleared with xylene and embedded in paraffin. Sections (5 μm in thickness) were prepared for H&E staining (Solarbio, G1120, Beijing, China) and Masson staining (Solarbio, G1340, Beijing, China). The operation was performed in strict accordance with the instructions provided for the kit.

### 4.5. Quantitative Real-Time PCR

TRIzol reagent (Invitrogen) was used to extract total RNA from liver tissues and cells. Then RNA concentration was measured using spectrophotometer (Thermo Fisher Scientific, Waltham, MA, USA). A PrimeScript™ RT reagent kit (TaKaRa, RR047A, Shiga, Japan) was used to synthesize cDNA, and PCR was carried out with a SYBR Premix Ex Taq II kit (TaKaRa, RR820A) according to the manufacturer’s instructions. Results were examined through the 2^−ΔΔCt^ method. The sequences of the primers were as follows (Table 1).

### 4.6. Western Blot Analysis

The extraction of total proteins was performed using RIPA buffer, which included protease and phosphatase inhibitors (Roche, Basel, Switzerland). Proteins were resolved by 10% SDS-PAGE subsequently and transferred to nitrocellulose membranes, blocked with 5% skim milk for 1 h, and incubated with primary antibodies overnight at 4 °C. After washing with TBST, membranes were then incubated with secondary antibodies for 1 h and visualized with the Odyssey Imaging System (Odyssey CLX, Biosciences, San Jose, CA, USA). The antibodies used in this experiment included anti-collagen I (1:500, WanLei, Shenyang, China) and anti-GAPDH (1:1000, Proteintech, Wuhan, China).

### 4.7. EdU Assay

Cell proliferation was detected by 5-ethynyl-2′-deoxyuridine (EdU) fluorescence staining according to the manufacturer′s instructions for the Cell-Light EdU DNA cell proliferation kit (RiboBio, Guangzhou, China).

### 4.8. Apoptosis Assay

Cells were seeded in 6-well plates, and after each group was treated according to the experimental scheme, the cells were washed twice with cold PBS and centrifuged at 1000 rpm for 5 min. The cell suspension was then mixed with 5 μL of Annexin V-FITC solution and nurtured for 15 min to avoid the sun. Afterward, 5 μL of PI solution was added for 5 min. Finally, 300 μL of 1× binding buffer was added to every group, and the apoptosis rate was detected within 1 h.

### 4.9. Immunofluorescence Analysis

HSC-T6 cells in the logarithmic growth phase were inoculated in 12-well plates and rinsed three times with PBS before being fixed with 4% paraformaldehyde for 15 min. The cells were then permeabilized with 0.1% Triton-X100 for 1 h and blocked with 5% BSA for 30 min. The cells were subsequently incubated with primary antibody (anti-α-SMA, 1:500, Abcam, Cambridge, UK) overnight at 4 °C. After being washed with PBS, the cells were incubated with secondary antibodies for 1 h. Finally, the nuclei were stained with DAPI. Immunofluorescence was analyzed under a confocal microscope (ZEISS, Jena, Germany).

### 4.10. Migration Assay

Cells were inoculated in a 6-well plate, and 3 parallel lines were drawn on the back of the 6-well plate in advance. Then, a sterile pipette tip (200 μL) was applied to scratch horizontal lines perpendicular to the back. Images were obtained at 0 and 24 h after scratching to assess cell migration, and the migration distance was calculated. The cell migration rate was quantified using the following equation: (area of initial scratch − area of current scratch)/area of initial scratch × 100%.

### 4.11. Transwell Assay

Cells were seeded in a 24-well Transwell plate, with the insert as the upper chamber and the well as the lower chamber. After detachment in serum-free medium, the cell suspension was adjusted to 2 × 10^4^ cells/mL and added to the upper chamber. The lower chamber was filled with DMEM containing 10% FBS. Following incubation, cells were fixed with 4% paraformaldehyde for 20 min and stained with 1% crystal violet (Solarbio, Beijing, China), after which representative images were obtained. Five nonrepeating fields per chamber were selected for imaging and counting.

### 4.12. Monodansylcadaverine Staining

The autophagy rate of the cells was detected by MDC staining. All operations were performed in accordance with the product instructions. Briefly, HSC-T6 cells were suspended and washed with wash buffer and resuspended again, after which they were stained with MDC staining solution for 15–30 min in the dark. After being washed with wash buffer, the cells were then observed and photographed under a fluorescence microscope. The MDC kit was purchased from Solarbio Biotechnology (China).

### 4.13. Statistical Analysis

All the experiments were performed at least three times and data were presented as mean ± SD. Statistical analysis was carried out with GraphPad Prism version 8.0.2 software. Significant differences between the two groups were tested by unpaired *t*-test. Differences were assessed by one-way ANOVA across three or more groups, followed by post hoc Tukey’s test for multiple comparisons. *p* < 0.05 was considered statistically significant.

## 5. Conclusions

In this study, we found that total flavonoids from *Carthamus tinctorius* L. (TFCTLs) exerted excellent anti-liver fibrosis effects in a dose-dependent manner. Further mechanistic investigation revealed that TFCTLs could inhibit the Hedgehog (Hh) signaling pathway, subsequently activating autophagy, which in turn attenuated hepatic stellate cell activation and proliferation while promoting apoptosis. These findings highlight the potential of TFCTLs as a promising therapeutic agent for liver diseases.

## Figures and Tables

**Table 1:** Sequences of the primers.

Species	Gene Bank No.	Gene	Forward Primer	Reverse Primer
Mouse	NM_007392.3	*α-SMA*	GGCTCTGGGCTCTGTAAGG	CTCTTGCTGTGGGCTTCATC
	NM_007742.4	*Collagen I*	GCTCCTCTTAGGGGCCACT	CCACGTCTCACCATTGGGG
	NM_009864.3	*E-cadherin*	AACCCAAGCACGTATCAGGG	GAGTGTTGGGGGCATCATCA
	NM_011701.4	*Vimentin*	ACCATCGCGGCTAAGAACAT	GCGGGCCATCTCATCCTTTA
	NM_001359819.1	*Beclin1*	ATGGAGGGGTCTAAGGCGTC	TCCTCTCCTGAGTTAGCCTCT
	NM_001364358.1	*LC3*	TTATAGAGCGATACAAGGGGGAG	CGCCGTCTGATTATCTTGATGAG
	NM_176996.5	*SMO*	AGTTGCTGCTGCTGGTACTG	GTGCAACGCAGAAAGTCAGG
	NM_010296.2	*Gli1*	GGTCTCGGGGTCTCAAACTG	TGTAGTGCTGAGCAGGTGTG
	NM_001289726.2	*GAPDH*	AGGTCGGTGTGAACGGATTTG	GGGGTCGTTGATGGCAACA
Rat	NM_031004.2	*α-SMA*	CATCCACGAAACCACCTA	GGGCAGGAATGATTTGGA
	NM_053304.1	*Collagen I*	TGTTGGTCCTGCTGGCAAGAATG	GTCACCTTGTTCGCCTGTCTCAC
	NM_031334.1	*E-cadherin*	TCTCTTGTCCCTTCCACAGC	CTCCAGACCCACACCAAAGT
	NM_031140.1	*Vimentin*	TGAGATCGCCACCTACAGGA	AAGGTCATCGTGGTGCTGAG
	NM_001034117.1	*Beclin1*	GAATGGAGGGGTCTAAGCCG	CTTCCTCCTGGCTCTCTCCT
	NM_022867.2	*LC3*	GAAGACCTTCAAACAGCGCC	CTTGGTCTTGTCCAGGACGG
	NM_012807.2	*SMO*	GTCACTCCCCTGTCCTCAC	GCACGGTATCGGTAGTTCTTGT
	NM_001191910.2	*Gli1*	AACATGGCAGTCGGTAACATGAG	CCGCGTGTGTGTAGCCATTTAG
	NM_017008.4	*GAPDH*	CACCATCTTCCAGGAGCGAG	CTCGTGGTTCACACCCATCA

## Data Availability

The data presented in this study are available on request from the corresponding authors due to privacy.

## Abbreviations

α-smooth muscle actin (α-SMA); carbon tetrachloride (CCl_4_); extracellular matrix (ECM); hepatic fibrosis (HF); hepatic stellate cells (HSCs); hydroxysafflor yellow A (HSYA); smoothened (SMO); the Hedgehog (Hh) signaling; total flavonoids from *Carthamus tinctorius* L. (TFCTLs); transforming growth factor-beta 1 (TGF-β1); 3-methyladenine (3-MA); purmorphamin (PUR); traditional Chinese medicine (TCM); nonalcoholic fatty liver disease (NAFLD); patched (Ptch); smoothened (SMO); myofibroblasts (MFBs).
